# A Novel Bimodal Imaging Agent Targeting HER2 Molecule of Breast Cancer

**DOI:** 10.1155/2018/6202876

**Published:** 2018-03-06

**Authors:** Wei Lv, Yamei Shen, Hengli Yang, Rui Yang, Wenbin Cai, Jian Zhang, Lijun Yuan, Yunyou Duan, Li Zhang

**Affiliations:** ^1^Department of Ultrasound Diagnosis, Tang Du Hospital, Fourth Military Medical University, Xi'an, Shaanxi 710038, China; ^2^State Key Laboratory of Cancer Biology, Department of Biochemistry and Molecular Biology, Fourth Military Medical University, Xi'an, Shaanxi 710032, China

## Abstract

Nanobubble (NB), a newly developed nanoscaled ultrasound contrast agent (UCA) for molecular imaging, has been widely researched for these years. Targeting it with functional molecule, nanobubble can adhere selectively to cellular epitopes and receptors outside the vasculature via enhanced permeability and retention (EPR) effect of tumor blood vessel. To enhance the targeting rate of our previous prepared NBs-Affibody for HER2 (+) breast cancer imaging, we introduced a near-infrared fluorescent (NIRF) dye, IR783, in this study to enhance tumor-specific targeting rate and provide a promising modality for dual-mode imaging. The prepared IR783-NBs-Affibody presented a uniform nanoscale size around 482.7 ± 54.3 nm, good biosecurity, and stability over time. The encapsulation efficiency (EE) of IR-783 was 15.09% in the conjugates leading to a successful NIR fluorescence and ultrasound enhancement imaging ex vivo. IR783-NBs-Affibody was able to automatically accumulate on BT474 cells with a highly increased targeting rate of 85.4% compared with previous NBs-Affibody of 26.6%, while Affibody-guided HER2 binding was only found in HER2-positive cell lines (BT474 and T-47D). The newly developed IR783-NBs-Affibody is characterized with favorable HER2 targeting ability and bimodal imaging capability for breast cancer. Thus, IR783-NBs-Affibody holds great potential in molecular diagnosis for patients with breast cancer.

## 1. Introduction

The overexpressed HER2 receptor is a valuable biomarker for breast cancer as it accounts for about 15–20% for those patients who have been diagnosed with breast cancer. It is also the only one that has improved the clinical management for patients with HER2 (+) by using anti-HER2 targeted agents, such as trastuzumab, lapatinib, and pertuzumab [1]. HER2 protein overexpression is measured by immunohistochemistry or by fluorescence in situ hybridization (FISH) test nowadays [2]. According to the published joint guideline recommendations in 2007 of the College of American Pathologists (CAP) and the American Society of Clinical Oncology (ASCO), it has been a routine pathological diagnosis to detect HER2 status in breast cancer all over the world [3, 4], not only for the prognostic evaluation for breast cancer patients but also a key element of the adjuvant therapy for HER2-positive patients. However, tumor heterogeneity may have been responsible for the false-negative finding in limited biopsy samples both in primary tumor and axillary lymph nodes (ALNs). Thus, a more convictive method for comprehensive assessment of HER2 status of breast cancer is needed.

Imaging modalities, especially, molecular imaging, may provide more precise *in vivo* information through a noninvasive approach, which would improve the understanding of pathological behavior of tumor at different therapy courses. Among all these imaging methods, ultrasound is well known for its nonradiation, time and cost saving, real-time imaging patterns, and so on. Microbubbles, or ultrasound contrast agent (UCA), such as SonoVue, applied as blood pool agents with microscaled diameters [5, 6] have been adapted for molecular imaging within blood vessels in many research works. These modified agents cannot leak outside the tumor vasculature to target a specific tumor-expressing molecule directly because of the agent size, which hampers the truly molecular imaging by ultrasound.

In our previous studies, we have fabricated a new nanosized UCA nanobubble (NB) through a revised thin-filmed hydration method [7] and targeted NB with a small HER2 antibody, Affibody [8]. The results demonstrated a high HER2-specific binding ability of NB-Affibody conjugates for HER2-positive breast cancer both ex vivo and *in vivo*. However, considering a limited targeting rate around 20% and a comparatively poor ALN imaging capability for UCAs, in this study, we introduced another near-infra fluorescent dye as we tried previously [9], IR783, which has been approved for a tumor-targeting selectivity and enhanced new bubbles' targeting rate as well as a higher sensitivity for sentinel lymph node (SLN) mapping due to their low background autofluorescence. Besides, bimodal imaging modalities providing both optical and ultrasound imaging are complementary and will offer more diagnostic information for patients with breast cancer.

## 2. Materials and Methods

### 2.1. Materials

DPPC (phospholipids 1,2-dipalmitoyl-sn-glycero-3-phosphocholine; Mw = 734 g/mol) and DSPE-PEG-Biotin (2000) (1,2-distearoyl-sn-glycero-3-phosphoethanolamine-N-[biotinylated(polyethyleneglycol)-2000]; Mw = 3017 g/mol) were used for preparing nanobubbles. Both were purchased from Avanti Polar Lipids Inc. (Alabaster, AL). Other materials were obtained as followed: IR783 from Sigma-Aldrich (St. Louis, MO), octafluoropropane (C3F8) gas from the R&D Center (Beijing, China), ImmunoPure® streptavidin from Pierce (Rockford, IL), and biotinylated anti-ErbB2 Affibody® Molecule from Abcam (USA).

### 2.2. Preparation of the IR783-NBs-Affibody

In a dry 25 mL rotary evaporation bottle, 10 mg DPPC and 4 mg DSPE-PEG-Biotin (2000) were mixed and 2 mL chloroform was added to dissolve the mixture. Then, 200 *μ*L NIRF agent IR783 liquor (dissolved in chloroform, 1 mg/mL) was added. In a rotary evaporator (New Brunswick Scientific, New Brunswick, NJ), rotary evaporation was conducted at 120 rpm and 55°C. After 10 minutes, chloroform was completely evaporated and a uniform light green phospholipid thin film was developed. Subsequently, 1.5 mL hydration liquid (10% glycerol and 90% 1x PBS, *v*/*v*) was used for the film hydration. The bottle was put into an incubator-shaker (New Brunswick Scientific, NJ) at 130 rpm under 37°C for 60 min, then the suspension of IR783-loaded liposomal film was prepared. The suspension was transferred into a tube. After the air in the tube was removed and C_3_F_8_ gas was inflated, the tube was placed in a mechanical oscillator (Ag and Hg mixer, Xi'an, China) for 90 s to produce bubbles. To combine biotinylated anti-ErbB2 Affibody molecules with IR783-loaded NBs, the avidin-biotin method was applied. To prevent fluorescence quenching, tin foil was used to cover all bottles and tubes. In the control group, we used SonoVue. Finally, the IR783-NBs-Affibody was sterilized by CO60 irradiation for 30 min.

### 2.3. Measurement of IR783-NBs-Affibody Characteristics

1x PBS (10 mL) was used to dilute IR783-NBs-Affibody mother liquor (1 mL). The size distributions of IR783-NB-Affibody (1.5 mL) and SonoVue microbubble (1.5 mL) were measured by particle size analyzer (DelsaNano, Beckman Coulter, USA) at 25°C. The results were calculated as a mean value after three repeated measurements.

### 2.4. Morphologic Observation of IR783-NBs-Affibody

To further investigate the IR783-NBs-Affibody structure, transmission electron microscopy (TEM, FEIT12, America) was applied. SonoVue was also examined as control. A copper grid covered with amorphous carbon was used to dry the samples for 30 min before observing.

### 2.5. Efficiency of Encapsulation (EE) and Loading Rate of IR783 in IR783-NBs

The amount of the IR783 in IR783-NBs was determined by standard curve of UV–vis absorption spectra measured by a UV–vis spectrophotometer (Mapada UV-6100S, Shanghai, China). IR783-NBs was separated from the original solution by centrifugation of 1500 rpm for 5 min at 4°C. All the agents, IR783, DPPC, DSPE-PEG-Biotin, and IR783-NBs, were dissolved in ethyl alcohol. The encapsulation efficiency and loading rate of IR783 were calculated as follows:

(1) $$\begin{array}{c} EE\left(\%\right)=\frac{weight\text{ of }IR783\text{ in }IR783-NBs}{weight\text{ of }total\;added\;IR783}\times 100\%,\\ IR783\;loading\;rate\left(\%\right)=\frac{weight\text{ of }IR783\text{ in }IR783-NBs}{weight\text{ of }IR783-NBs}\times 100\%. \end{array}$$

### 2.6. Biosecurity of IR783-NBs-Affibody

BT-474 human breast cancer cells were cultured in Dulbecco's modified Eagle's medium (DMEM) with 10% fetal bovine serum (FBS) and placed in a humidified atmosphere of 5% CO_2_ at 37°C. Then, BT-474 cells were seeded at a density of 5000 cells/well in 96-well plate. The cells were cultured for 24 h till they adhered to bottom. Subsequently, the cells were cultured with various concentrations of IR783 (1–40 *μ*g/mL) in fresh medium for 24 h. Then 100 *μ*L of fresh medium with 10 *μ*L CCK-8 was added to culture these cells for 4 h. The absorbance at 450 nm of each well was measured after the plate was oscillated at low speed for 30 s in a Microplate Reader (iMark, Bio-Rad, America).

### 2.7. NIRF and Ultrasound Bimodal Imaging of IR783-NBs-Affibody In Vitro

Optical imaging capability of IR783-NBs-Affibody was observed by laser scanning confocal microscopy (LSCM, Nikon A1R, Japan) with excitation at 640 nm and peak emission at 780 nm to obtain NIR fluorescent.

To complete ultrasound contrast imaging, a latex glove fingertip filling with 10 mL of 1x PBS was put in water at 25°C. MyLab Twice ultrasound system (Esaote, Italy) was used to acquire ultrasound images with a 5 MHz transducer. After the ultrasound images of PBS-containing glove can be acquired clearly, SonoVue and IR783-NBs-Affibody (100 *μ*L) were injected into PBS, respectively, then the ultrasound pictures were compared.

### 2.8. Stability of IR783-NBs-Affibody

A 10 *μ*L solution of IR783-NBs-Affibody was dissolved in 1 mL 1x PBS in a tube at 37°C. The change of the IR783-NBs-Affibody in the size distribution was measured for 20 min at every 5 min interval using the particle size analyzer. The diluted IR783-NBs-Affibody was exposed at 37°C for 20 min, and hemocytometer was used to calculate the number at 1, 5, 10, 15, and 20 min. All the abovementioned measurements were performed in triplicate.

### 2.9. Tumor-Specific Targeting of IR783-NBs-Affibody *In Vitro*

Three breast cancer cell lines (HER2-positive cells BT-474 and T-47D and HER2-negative cell MDA-MB-231) were incubated in the same condition as previously mentioned. When the BT-474 cells grew up to 70–80% confluence, all cells were trypsinized, divided into sterile four tubes (marked 1–4) with the same cell number, and centrifuged to remove culture medium. PBS, NBs, NBs-Affibody, and IR783-NBs-Affibody were added into tubes 1 to 4, respectively, at room temperature for 0.5 h. Then, the cells were washed with PBS for three times by centrifugation (Beckman Coulter, Fullerton, CA) before flow cytometry (FCM) analysis, the same operations for MDA-MB-231.

In a 20 mm confocal dish, after the BT-474 and MDA-MB-231 cells were attached to the bottom, the medium was discarded and the cell was washed with sterile 1x PBS lightly. NBs, NBs-Affibody, and IR783-NBs-Affibody (200 *μ*L, 6.0 × 10^6^/mL) were added to the dish, respectively, and coincubated at room temperature for 0.5 h. After being washed with PBS for three times, the cells were incubated with 4% formaldehyde (1 mL) for 15 min and kept at 4°C, then washed for three times. Subsequently, 0.1% Triton X-100 (1 mL) was added to enhance the penetration of substrate at room temperature for 10 min and washed away. Next, 1 *μ*g/mL DAPI (4′,6-diamidino-2-phenylindole) was added for nucleus staining and after 5-minute incubation, it was washed with 1x PBS. Fluorescence images were captured by LSCM with a ×20 objective lens. Each fluorescent was obtained at different wavelengths, for example, DAPI blue fluorescent was obtained at 405 nm for excitation and 475 nm for emission, DiO green fluorescent was obtained at 488 nm for excitation and 530 nm for emission, and IR783 NIR fluorescent was obtained at 640 nm for excitation and 780 nm for emission. HER2 targeting ability of IR783-NBs-Affibody was further assessed with FCM by both DiO and IR783 fluorescence detection in HER2 (+)/HER2 (−) cell lines. All the above procedures were conducted in dark three times.

## 3. Results

### 3.1. Size Distribution and Morphology of IR783-NBs-Affibody

**Figure 1:**
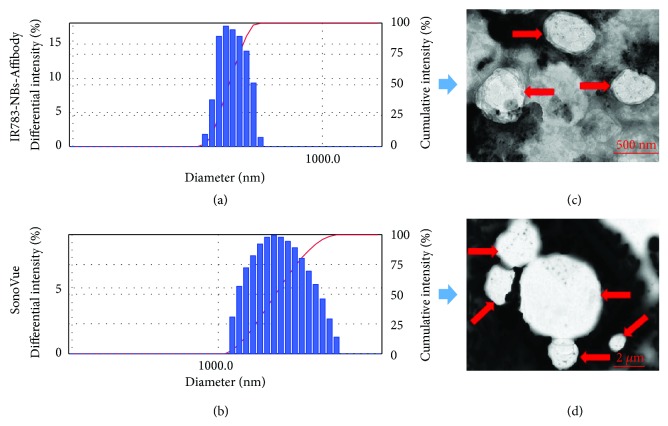
Comparisons of IR783-NanoBubble-Affibody and SonoVue in size distribution and morphology. Size distribution of IR783-NBs-Affibody (a) and SonoVue microbubbles (b). TEM images of IR783-NBs-Affibody (c) and SonoVue microbubbles (d).

The mean diameters of IR783-NBs-Affibody and SonoVue microbubbles were 482.7 ± 54.3 nm and 1784.7 ± 400.4 nm, respectively. The polydispersity index (P.I.) was 0.3107 ± 0.068 (*n* = 3) and 0.280 ± 0.036 (*n* = 3) accordingly (Figures 1(a) and 1(b)).

IR783-NBs-Affibody and SonoVue microbubbles obviously appeared spherical under TEM scanning. The size of SonoVue was over 1000 nm, while IR783-NBs-Affibody showed to be around 500 nm (Figures 1(c) and 1(d)). The results of particle size analyzer and TEM were consistent.

### 3.2. IR783 Loaded in IR783-NBs and Its Biosecurity

**Figure 2:**
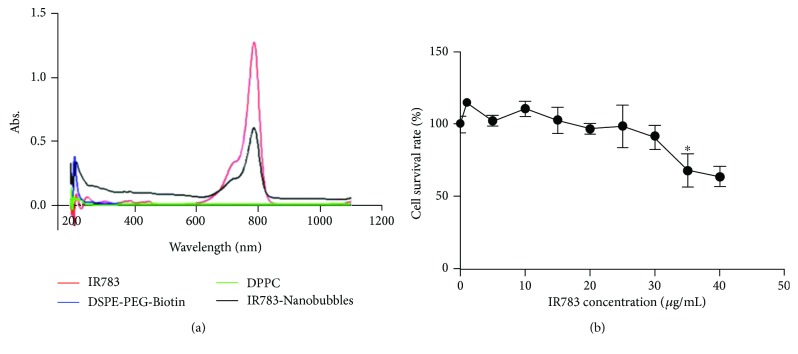
IR783-loaded NBs (IR783-NBs) and its biosecurity. (a) Absorbance spectra were measured in IR783, DPPC, DSPE-PEG-Biotin, and IR783-NBs in ethyl alcohol. The adsorption peak of 200 nm wavelength is caused by DSPE-PEG-Biotin and DPPC. (b) Cytotoxicity of different concentrations of IR-783 in BT-474 cells, as examined by a CCK-8 assay.

The EE of IR783 in IR783-NBs was about 15.09%, and loading rate was 0.21%. IR783 (3 *μ*g/mL in ethyl alcohol) exhibited a strong absorption peak at 786 nm, while DPPC and DSPE-PEG-Biotin showed absorption about 200 nm wavelength and no absorption in near-infrared region from UV–vis spectra. After being loaded with IR783, IR783-NBs appeared to have the same absorption peak at 786 nm as IR783 does without shift (Figure 2(a)).

Cell survival rate curve was obtained via cell counting kit (CCK-8) assay (Figure 2(b)). Obvious cytotoxicity was not observed until IR783's concentration was increased up to 35 *μ*g/mL, indicating that IR783 had no obvious cytotoxicity to BT-474 cells at a normal concentration (<5 *μ*g/mL) for research and clinical purposes.

### 3.3. Bimodal Imaging of IR783-NBs-Affibody *In Vitro*

**Figure 3:**
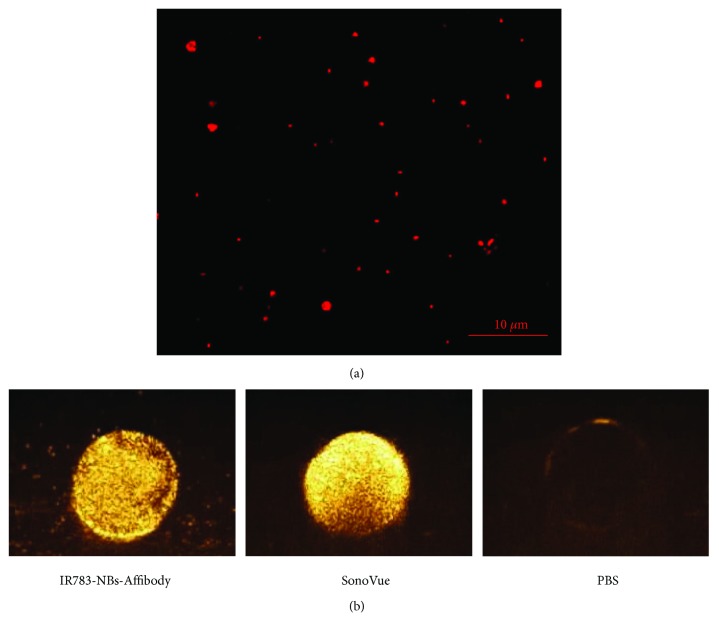
NIRF and ultrasound bimodal images of IR783-NBs-Affibody *in vitro*. (a) A LSCM image of IR783-NBs-Affibody. (b) The contrast-enhanced ultrasound images of IR783-NBs-Affibody, SonoVue microbubbles, and PBS *in vitro*, respectively.

Fluorescence inspection of IR783-NBs-Affibody was performed under LSCM scanning. As shown in Figure 3(a), many bubbles smaller than 1 *μ*m were clearly observed against a dark-field background and IR783 fluorescence was detected on the nanoscaled bubbles.

Compared with the negative control, IR783-NBs-Affibody exhibited significantly enhanced ultrasonic contrast ability as SonoVue MBs did (Figure 3(b)).

### 3.4. The Stability of IR783-NBs-Affibody

**Figure 4:**
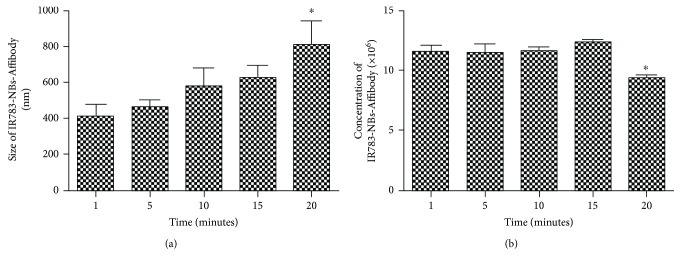
Stability of IR783-NBs-Affibody. (a) The changes of IR783-NBs-Affibody's size distribution over 20 min. ^∗^*P* < 0.05, obviously different from ones at 1 min. (b) The changes of IR783-NBs-Affibody's concentration over 20 min. ^∗^*P* < 0.05, obviously different from ones at 1 min.

The stability of IR783-NBs-Affibody was evaluated by two physical parameters including size distribution and concentration. The size distribution of IR783-NBs-Affibody was not significantly changed after 15 min compared to that of IR783-NBs-Affibody at 1 min (627.4 ± 125.5 nm versus 461.5 ± 113.2 nm, *P* > 0.05; Figure 4(a)). Nevertheless, the IR783-NBs-Affibody at 20 min changed into bigger bubbles obviously different from the ones at 1 min (811.0 ± 234.4 nm versus 461.5 ± 113.2 nm, *P* < 0.05; Figure 4(a)). Similarly, the concentration of IR783-NBs-Affibody was kept stable at 15 min compared with that at 1 min (12.3 ± 0.4 × 10^6^/mL versus 11.5 ± 0.8 × 10^6^/mL, *P* > 0.05; Figure 4(b)), while the concentration of IR783-NBs-Affibody was significantly decreased after 20 minutes (9.3 ± 0.6 × 10^6^/mL at 20 min versus 11.5 ± 0.8 × 10^6^/mL at 1 min, *P* < 0.05, Figure 4(b)).

### 3.5. The Tumor-Specific Targeting of IR783-NBs-Affibody

**Figure 5:**
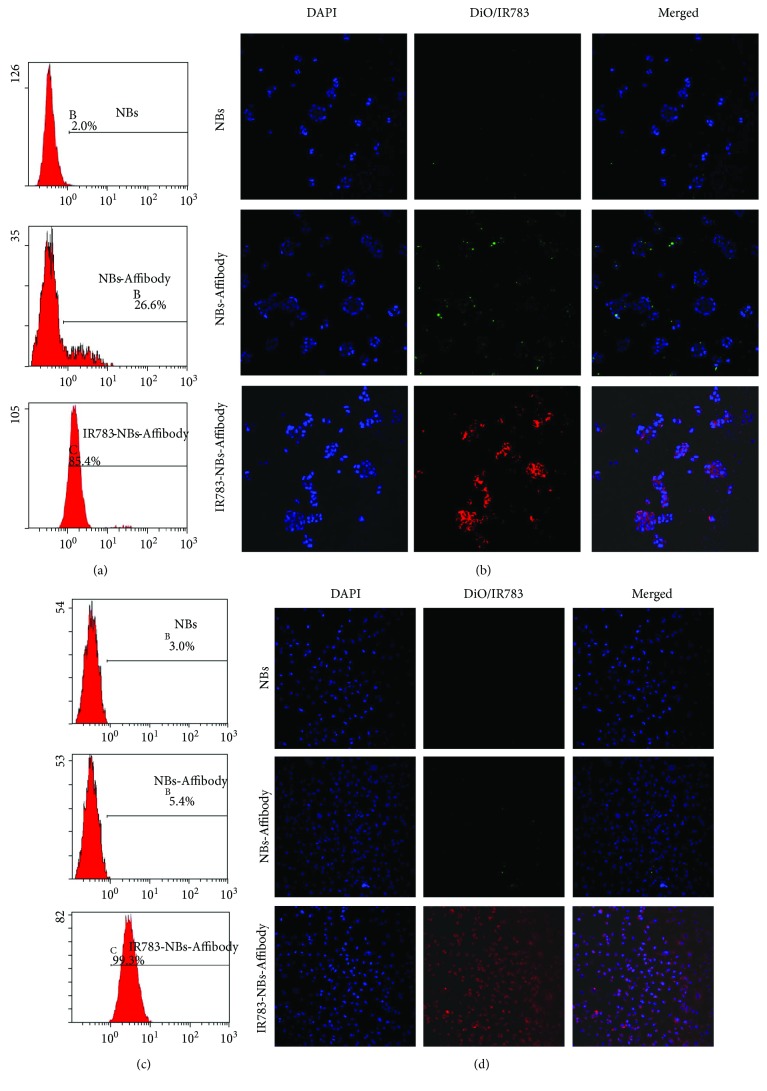
The tumor-specific targeting of IR783-NBs-Affibody towards HER2 (+)/(−) breast cancer cells. (a, c)The targeting rate of NBs, NBs-Affibody, and IR783-NBs-Affibody for HER2 (+) breast cancer cells (BT-474) and HER2 (−) breast cancer cells (MDA-MB-231) via flow cytometry. (b, d) LSCM pictures of BT-474 and MDA-MB-231 cells coincubated with NBs, NBs-Affibody, and IR783-NBs-Affibody with a ×20 objective lens.

The tumor-specific targeting rate towards HER2 (+) breast tumor cells BT-474 was increased greatly after IR-783 was introduced in the bubble from 26.6% of NBs-Affibody to 85.4% of IR783-NBs-Affibody (Figure 5(a)). For HER2 (−) breast tumor cells MDA-MB-231, the tumor-specific targeting rate was also increased from 5.4% of NBs-Affibody to 99.3% of IR783-NBs-Affibody (Figure 5(c)). LSCM images also confirmed that an accumulation of NBs-Affibody fluorescence on BT-474 cells was observed compared with NBs (Figure 5(b)) and with NBs-Affibody towards MDA-MB-231 without green fluorescence (Figure 5(d)). NIR fluorescence from IR783-NBs-Affibody was shown around almost all cells, and the amount of fluorescence accumulation in IR783-NBs-Affibody was much higher than that of NBs-Affibody in both BT-474 and MDA-MB-231 cells.

**Figure 6:**
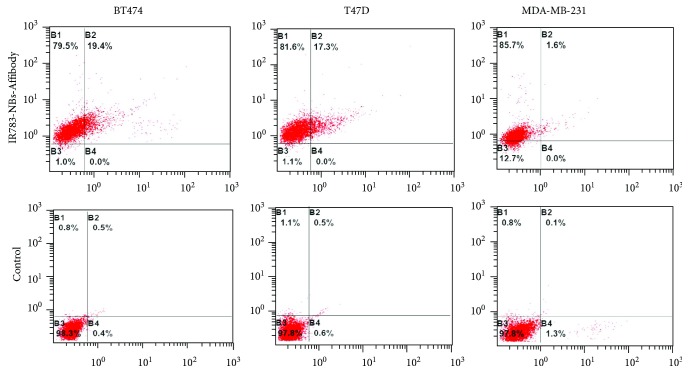
The HER2-specific targeting rate of IR783-NBs-Affibody towards HER2 (+) breast cancer cells BT-474/T-47D and HER2 (−) breast cells MDA-MB-231. The B1 area represents cells dyed with IR783, B2 for cells dyed with both IR783 and DiO, B3 for cells without fluorescence, and B4 for cells dyed with DiO.

Further fluorescence detection demonstrated that only IR783-NBs-Affibody showed both tumor-specific and HER2-specific binding abilities towards HER2 (+) tumor cells. The HER2 targeting rate of IR783-NBs-Affibody was kept at 19.4% and 17.3% for BT-474 and T-47D cells, respectively (Figure 6).

## 4. Discussion

The major achievement of this study is to prepare a new nanobubble that can be served as a bimodal imaging agent in both contrast-enhanced ultrasound (CEUS) and optical imaging for patients with breast cancer to identify both primary tumor and small metastasis and also determine the expression status of HER2 within tumor. As we know, the majority of patients can be diagnosed with breast cancer at early stage with the development of modern imaging technologies nowadays. For those patients, breast conserving therapy (BCT) is recommended [10, 11]. Identification of surgical margin and ALN involvements may enhance clinical preoperative decision-making for BCT. Although intraoperative ultrasound (IOUS) and CEUS further increase the capability of localizing the surgical margin and potential ALNs, many questions still remain. Without tumor or tumor subtype molecule targeting, US and CEUS are incapable of judging the malignant margin or nodes from benign ones [12–14]. In previous studies, HER2-targeting nanobubbles showed promise in HER2 targeting ability both *in vitro* and *in vivo* [8]. However, the HER2 status varies between a primary and metastatic site [15]. A total of 13% of cancers that were HER2 (+) in primary cancer were found to be HER2 (−) in metastatic lesion, whereas 5% to be negative in the primary tumor were positive in metastatic specimen in a meta-analysis [16]. Tumor heterogeneity may have been responsible for the negative finding in the biopsy sample. Thus, an over 20% HER2-binding rate of NBs-Affibody that has been observed may not be sufficient for a promising signal-to-noise ratio (SNR) imaging, which depends on the contrast agent's biodistribution to acquire a signal strength comparison between the targeted tissue and the surrounding area.

IR783, as well as IR-780 iodide and MHI-148, has been demonstrated with preferential accumulation in cancer tissues by itself with no need of conjugation to guiding moieties [17, 18]. Despite of the unclear mechanism of the cyanine dyes' specific accumulation and retention, some researches have illustrated that the implications of EPR effect, organic-anion transporting polypeptides (OATPs), and mitochondrial membrane potential might have some relation [19, 20]. However, because of the existing lipophilic profile, NIRF dyes are extremely difficult to dissolve in water, which hampers their clinical utilization greatly. Nowadays, interests have been proposed in improving the bio-compatibility of NIR fluorescence dyes by reengineering them with different carriers for cancer diagnosis and therapy [21, 22]. Among these carriers, phospholipid-shell and gas-core NBs exhibited ideal compatibility in encapsulating NIR dyes [6, 23–25]. Our study confirmed a successful conjugation of IR783 with NBs with the EE about 15.09% through simple loading instead of chemical conjugation. The EE which was low might be the reason of the processing procedure that IR783-NBs was separated from the original solution by centrifugation of 1500 rpm for 5 min at 4°C. It is difficult to obtain all IR783-NBs after centrifugation because the stability of IR783-NBs would be challenged by bubble-burst and some IR783-NBs became adhesive to the wall. Although the EE and loading rate of IR783 were low, IR783-NBs-Affibody presented optimal tumor targeting towards HER2 (+)/(−) breast cancer cells via FCM and LSCM tests.

As our results indicated that by introducing IR783, an amphiphilic molecule with NIRF imaging and tumor-targeting capabilities into previous NBs-Affibody, the newly prepared IR783-NBs-Affibody, showed a much higher tumor-specific binding ability to BT474 cells and MDA-MB-231 higher than 80% compared with previous NBs-Affibody. NIR fluorescence was accumulated around most of the breast cancer cells as detected by LSCM in IR783-NBs-Affibody group. The passive targeting through EPR effect, HER2 binding ligand, and IR783 tumor auto-selectivity was enhanced greatly. The targeting rate of IR783-NBs-Affibody was found to be similar either to HER2-positive (BT-474 and T-47D) or to HER2-negative (MDA-MB-231) cells. HER2-labelled fluorescent cells were detected only in HER2-positive cell lines. The reason might be that IR783-mediated tumor binding is a kind of tissue-specific targeting in differentiating tumor tissue from normal tissue, but not a specific molecule binding for further tumor subtype imaging, even without the tumor subtype recognition unit. Thus, by integrating anti-HER2 monoclonal antibody and heptamethine dye IR783 into the nanoscaled UCA, the IR783 function of IR783-NBs-Affibody enables the tumor-specific recognition for primary tumor and possible metastasis, while the Affibody function determines the HER2 expression. More interestingly, the binding rate of IR783-NBs-Affibody to HER2-positive tumor cells varies depending on the variability of HER2 expression on different cell lines. According to Chen et al.'s report that BT474 cells express HER2 higher than T-47D cells do [26], we detected more HER2-labelled cell group in the present study. This finding can be applied in quantitative diagnosis of HER2 expression level in further studies.

Imaging with high spatial resolution is urgently required for accurate tumor margin delineation and localization of potential small metastases. Thus, bimodal or multimodal imaging agent has attracted increasing attention as it can provide a multitechnical complementation to achieve more imaging information about the lesion and also offer a potential agent-mediated strategy [27–29]. On the one hand, both imaging agents can be easily conjugated together in the preparation procedure and also showed favorable physical properties including uniform nanoscale size, good stability, and low cytotoxicity. On the other hand, the dual-modality imaging of optical and ultrasound would provide a complementary mode for tumor molecular diagnosis. Several studies reported that modified NIR fluorescence by conjugating various molecules showed excellent accuracy in the intraoperative detection of tumor margins [30, 31]. NIR fluorescence is also a good tracer in visualizing the lymphatics and lymph nodes [32–34] and has been explored for intraoperative image-guiding modality [34, 35] with diverse degrees of success. The preferential accumulation of NIR fluorescence in tumor can provide a significantly higher SNR in tumors [36, 37]. There are still drawbacks in optical imaging method because of the absorbing and scattering features of bio-tissue and rapid optical fluorescence declining with the depth increasing. Nevertheless, the ultrasonic scattering of UCA could help imaging agents to achieve high acoustic resolution at depths. Our result also indicated that IR783-NBs-Affibody showed similar echogenic ability with SonoVue *in vitro* because the compressible liposome membrane is apt to form and yield echogenicity allowing a high scattering of NBs under a low MI and harmonic imaging modes [38, 39]. Therefore, CEUS is compatible with optical imaging, thereby achieving bimodality imaging by a complementary contrast.

## 5. Conclusion

The obtained bimodal imaging agent IR783-NBs-Affibody providing both ultrasound and near-infrared fluorescence imaging signals is able to achieve tumor-specific and HER2-specific targeting simultaneously. IR783-NBs-Affibody presents nanoscale uniform size, a low polydispersity, and favorable stability. For ultrasound mode, IR783-NBs-Affibody shows a promising enhanced ultrasound signal *in vitro* similar to SonoVue does; while for NIR fluorescence mode, IR783-NBs-Affibody-treated tumor cells exhibit high fluorescence intensity. Thus, IR783-NBs-Affibody is expected to achieve a high SNR when two imaging modalities are applied in breast cancer patients, which is helpful for tumor delineation and possible SNL trace.
